# Effect of hyperglycaemia in pregnancy on adiposity in their infants in India: a protocol of a multicentre cohort study

**DOI:** 10.1136/bmjopen-2014-005417

**Published:** 2014-06-27

**Authors:** Giridhara R Babu, Lavanya Garadi, G V S Murthy, Sanjay Kinra

**Affiliations:** 1 IIPH-H, Bangalore Campus, Public Health Foundation of India, Bangalore, Karnataka, India; 2 IIPH Hyderabad, Hyderabad, Andhra Pradesh, India; 3 Faculty of Infectious and Tropical Diseases, Department of Clinical Research, Public Health Disability, London School of Hygiene & Tropical Medicine, London, UK; 4 Faculty of Epidemiology and Population Health, Department of Non-communicable Disease Epidemiology, London School of Hygiene & Tropical Medicine, London, UK

**Keywords:** Epidemiology

## Abstract

**Introduction:**

The carbohydrate ‘fuel’ metabolism in a pregnant woman may have a long-term impact on the development of her offspring (‘fuel-mediated teratogenesis’ hypothesis) including in utero exposure to maternal hyperglycaemia leading to fetal hyperinsulinaemia, and the consequent increase in fetal fat cells. Therefore, a feed-forward loop can exist of rising adiposity and hyperinsulinaemia throughout childhood, perhaps leading to obesity and diabetes in later life. There is a need for prospective examination of body fat distribution in children born to mothers with different glycaemic levels to understand the plausible association between glucose metabolism and future risk of diabetes in offspring. The hypothesis is that maternal glucose levels in pregnant women are related to skinfold thickness in their infants.

**Methods and analysis:**

Hyperglycaemia in pregnancy and adiposity in infants is a multicentre cohort study to evaluate the effects of glucose levels in pregnancy on the risk of adverse infant outcomes, especially in predicting the risk of chronic diseases in infants. The study aims to recruit 1045 participants over a period of 1 year, who will be followed up irrespective of their glycaemia status for a period of 15 months, beginning in the 24th week of gestation. The glucose levels in pregnant women would be obtained through oral glucose tolerance testing. The primary outcome of our study was skinfold thickness in infants at the age of 6 weeks, 3rd and 9th month (as a proxy for fat distribution/adiposity).

**Ethics and dissemination:**

The institutional review board at The Indian Institute of Public Health (IIPH)-Hyderabad, Public Health Foundation of India has approved the protocol. All participants are required to provide written informed consent.

## Introduction

India is dubiously termed as ‘the diabetes capital of the world’.^1^ The number of people with diabetes in India was estimated at 50.8 million in the year 2000 and is projected to be 70 million by the year 2025.^2^ Urbanisation-related lifestyle changes are clearly driving the current epidemics of obesity and diabetes in the developed and developing world alike,^1^ ^3^ but they fail to explain adequately the remarkable rate and magnitude of these epidemics.^4^ Accordingly, additional contributory mechanisms have been proposed. The ‘thrifty phenotype’ hypothesis suggests that the risks of obesity, diabetes and cardiovascular diseases are ‘programmed’ in early life through the persistence of endocrine, physiological and metabolic adaptations made in the face of undernutrition.^4^ ^5^ The ‘thrifty phenotype’ is disingenuous, in the sense that it confers an early survival advantage on the individual during undernutrition but can become deleterious under conditions of overnutrition in adulthood. Similarly, conservatory adaptations in the genotype of populations that have undergone rapid transition from episodic undernutrition to plentiful food supply results in alterations of genotype, referred to as the ‘Thrifty Genotype’ hypothesis. One such is the ‘thrifty (fetal) insulin hypothesis’, which proposes that low birthweight and type 2 diabetes mellitus (T2DM) are two phenotypes of the same genotype that also predicts insulin resistance.^6^ The ‘thrifty phenotype’ hypothesis is supported by a substantial body of research in animal models, and by observational studies.^7^

It is therefore intriguing to understand the mechanism that led to T2DM in populations. Evidence for the relationship between hyperglycaemia and adverse effects is stronger when studied between mothers and offspring (compared with fathers and offspring).^7^ ^8^ Furthermore, the pattern of birthweight diabetes association in high-risk populations, as shown in the pregnancy cohort from Mysore in south India,^9–11^ is often ‘U’ shaped. In this study, the excess prevalence of diabetes in the high birthweight group disappeared on adjusting for maternal diabetes, implying a maternal contribution to the link.^12–17^ It is therefore important to explore the persistence and sequelae of hyperglycaemia in pregnancy into T2DM in later life.^18^ The estimates of hyperglycaemia among pregnant women in India vary between 1.5% and 17.7%.^3^ ^19^ ^20^ This wide variation may be due to the application of several screening tests of varied quality and a lack of consensus practice of diagnostic criteria for hyperglycaemia in pregnancy. Part of the variability may also reflect differences in the distribution of genetic and environmental risk factors. Maternal obesity is a major and modifiable risk factor for hyperglycaemia in pregnancy,^21^ ^22^ although there are other non-modifiable factors such as genetic predisposition, age and ethnicity.^23^ The risks of overt hyperglycaemia in pregnant women to the health of the mother (greater risk of diabetes in later life)^24^ and to the offspring in the short term (adverse perinatal outcomes such as macrosomia, birth trauma and metabolic abnormalities) are well established.^25–28^ The probable consequences of hyperglycaemia in pregnant women can include changes in the intrauterine environment that might increase childhood obesity risk and further risk of T2DM.^29^ Literature reviews indicate that offspring of mothers with hyperglycaemia in pregnancy are at higher risk of obesity,^29^ ^30^ which may depend on the form and severity of maternal diabetes.^29^ ^31–34^ Despite this evidence, the risks associated with relatively less severe degrees of glucose intolerance during pregnancy are unclear. It is found that increasing maternal carbohydrate intolerance even in normal pregnant women can be associated with a graded increase in adverse maternal–fetal outcomes.^8^ ^22^

This continuum of slowly increasing risks corresponding to mounting blood glucose levels makes it onerous to make a distinction between an adverse effect of hyperglycaemia and its counterfactual physiological effect.^35^ Some part of this intricacy is solved by the possibility that carbohydrate ‘fuel’ metabolism in a pregnant woman may have a long-term impact on the development of her offspring (*‘*fuel-mediated teratogenesis’ hypothesis).^26^ This hypothesis proposes that in utero exposure to maternal hyperglycaemia is thought to result in fetal hyperinsulinaemia,^36^ ^37^ and the consequent increase in fetal fat cells. Therefore, this results in a feed-forward loop of rising adiposity and hyperinsulinaemia throughout childhood, perhaps leading to obesity and diabetes in later life.^36^ ^37^ The rise in the prevalence of adiposity and glucose intolerance is accompanied by a progressive lowering of the age at onset of these conditions. When this reaches a stage of substantial overlap with childbearing age among women, it triggers contributions to the epidemic from ‘fuel-mediated teratogenesis’. It is thought that the feed forward mechanism results in *transgenerational transmission* of adiposity and glucose intolerance in women.^38^ If the propositions incorporating the known hypotheses are true (see online supplementary file), there is optimism for a clear strategy for the control of the obesity–diabetes epidemic through rigorous screening and management of adiposity and glucose intolerance in pregnancy.

### Rationale

To broaden the plausible association between glucose metabolism and future risk of diabetes in offspring, there is a need for prospective examination of body fat distribution in children born to mothers with different glycaemic levels.^29^ ^39^ The hypothesis is that maternal glucose levels measured by glucose levels in pregnant women are related to skinfold thickness in their infants.

## Methods

Hyperglycaemia in pregnancy and adiposity in infants (HyPA) is a multicentre study to evaluate the effects of glucose levels in pregnancy on the risk of adverse infant outcomes, especially in predicting the risk of chronic diseases in infants. HyPA will apply prospective cohort study design to investigate the extent to which differing levels of glucose are associated with infant outcomes. HyPA will provide information to assist clinicians and policymakers in shaping guidelines for the management of pregnant women with differing levels of blood glucose.

### Setting

The field centres comprise public health facilities in the city of Bengaluru. Each of these facilities registers approximately 130–150 pregnant women for antenatal care every month. A majority of these pregnant women register for antenatal check-up (ANC) around the 16th week of pregnancy.^40^ Generally, a minimum of three ANCs is offered for the pregnant women and at least once a month in case of high-risk pregnancies. Located in a metropolitan city, these hospitals conduct some laboratory tests on a routine basis.

*Sample size*: we used STATA/SE V.10.1 for Macintosh (2008) to calculate the sample size for the HyPA study.^41^ The study is powered at 90% with α=0.05, β1 coefficient=0.09 and SD=0.5. The current study is planned as a precursor for a larger cohort study with an estimated 10 000 people. To test the feasibility of the large cohort, the HyPA study plans to recruit 1000 pregnant women. The power calculation reveals that for a two-sided 95% CI, normal approximation yields a power of 32 with α=0.05, β1 coefficient=0.09 and SD=0.5. The sample size for the planned large cohort study was calculated by normal approximation with continuity correction and the power was 90%. For the current pilot study, we took 10% of the total sample size required for the large study with a power of 32%.^42–47^

### Study population

The source population for the study will comprise all pregnant women approaching the study centres for ANC. The study will be carried out in three selected health centres in Bengaluru city. We aim to recruit around 350–375 pregnant women in each centre over the next year resulting in the desired sample size. The participation in the study is voluntary and the participants will be instructed regarding the steps in the study, including the adverse effects. The recruitment will be carried out based on the standard protocol and after obtaining informed consent from the pregnant women. The cohort study aims to recruit 1045 participants over a period of 1 year, who will be followed up irrespective of their glycaemia status for a period of 15 months, beginning in the 24th week of gestation. Adiposity levels in the offspring will be compared based on the glycaemic status of the mother.

*Participants:* The women will be recruited at or before 24 weeks of gestation. They will be recruited for the study after obtaining informed consent and relevant details will be noted on a predesigned data collection format*.*

The eligibility criteria are as follows:

The age of the woman should be ≥18 years and ≤40 years and gestational age should be ≥14 weeks and ≤24 weeks. The HIV test result should be negative and the woman should be accessible to the study team for the next 1 year, so she should agree to reside in the study area for at least a year.

The exclusion criteria are:

- Hypertension;
- Known diabetes;
- Heart disease;
- Any cancer or renal disease.
- In addition, conditions like:
  Thyroid diseases, tuberculosis
  Asthma
  Epilepsy
  Current/previous treatment for infertility will be excluded.
- Further, multiple (twin) intrauterine gestation or a history of four or more live births previously will also be a criterion for exclusion.

Age and education level will also be recorded for women who decline to participate. As per our protocol, all patients will undergo oral glucose tolerance testing (OGTT) at or after 24–28 weeks of gestation. Additionally, a glycated haemoglobin (HbA1c) test and cord blood specimens for insulin will be estimated in a subset of 300 pregnant women to compare the relative accuracy of all glucose estimations. In addition, birthweight and length of the baby will be measured at birth. The measurements on skinfold thickness and weight for length in infants will be recorded at 14 weeks and at 9–12 months of age.

### Consent procedures

All eligible women will be provided study information leaflets. They will be provided time to ensure that they understand the information and have the opportunity to clarify and ask questions. All eligible women will have the choice to participate or decline to participate in the study. Participants will be assured that their participation or non-participation in the study will not affect their clinical care. Written informed consent will be obtained from the women who wish to participate.

## Data collection

### Baseline

At the time of recruitment to the study, the following baseline information will be recorded:

- Demographic factors
- Nutritional assessment
- Demographic and social characteristics
- Nutritional assessment and dietary characteristics
- Physical activity assessment
- Social Support Assessment Scale
- Mental health assessment
- History of tobacco and alcohol intake
- Medical and obstetric history
- Skinfold thickness in pregnant women

### Follow-up

At the time of follow-up during the study, the following information will be recorded:

- Skinfold thickness in infants
- Nutritional assessment
- Delivery information and infant birth details
- Maternal anthropometry
- Newborn assessment details
- Infant anthropometry at 6 weeks, 3rd and 9th month
- Infant morbidity details
- Immunisation record
- Infant feeding details.

### Qualitative analysis

A purposive sample of 20 women will be invited to attend a semistructured interview to obtain a more detailed insight of their experiences. We will choose women of different age groups, social background and culture, with the aim of capturing information from a diverse population of women who attend the antenatal services at public health facilities.

## Measures

### Exposure assessment

The exposure of interest is a continuous range of glucose. We will follow the recommendations of the International Association of Diabetes and Pregnancy Study Groups (IADPSG).^48^ We will follow the WHO (1998) recommendations for OGTT, where respondents are instructed to not have any food after 21:00 on the day prior to the testing. All respondents who have consented to be tested will be asked to be available at the study centre by 9:00 and ensured that they have fasted for a minimum of 10–12 h. After collecting a fasting sample, 75 mg of anhydrous glucose will be administered orally, with 250–300 mL of water, within 5 min of collecting the sample. All respondents who have consented to be tested will be asked to be available at the study.

**Figure 1:**
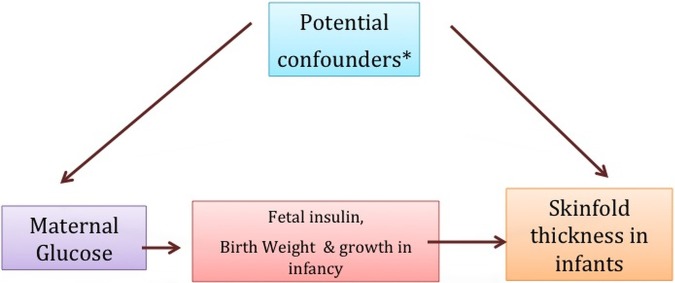
Directional acyclic graph depicting the hypothesis of hyperglycaemia in pregnancy and adiposity in infants.

We will also perform HbA1C in a small subsample of 100 pregnant women to compare with the values obtained by OGTT. All the samples will undergo external quality control check carried out by UK NEQAS for assuring global quality control. As an important mediator as explained (see figure 1), cord-blood specimens will be collected in a small subset at delivery for the measurement of serum C-peptide and plasma glucose levels.^49^ It has been documented that the cord-blood serum C-peptide level provides a better index of fetal β-cell function than does the insulin level.^49^ The reliability and validity scores will guide us in determining the most economical and efficient measure of maternal glucose levels for the larger cohort study.^49^

### Outcome assessment

The primary outcome of our study is skinfold thickness in infants at the age of 6 weeks, 3rd and 9th month (as a proxy for fat distribution/adiposity). Triceps, subscapular and triceps plus subscapular skinfolds will be measured. Height will be measured to the last 0.1 cm by standard methods. Weight will be recorded to the last 100 g. Triceps and subscapular thickness will be measured as described by Tanner and Whitehouse.^50^ The measurements will be carried out using Holtain calipers (Holtain, UK).^51^ The triceps skinfold will be measured over the posterior belly of the triceps muscle of the right arm, halfway between the acromion and the olecranon, on a line passing upwards from the olecranon in the axis of the limb, with the arm extended. Subscapular skinfold thickness will be measured immediately below the angle of the right scapula, in the natural cleavage line of the skin, with the arm held by the side of the body. Skinfold measurements will be performed by lifting the full thickness skin with the thumb and index finger, with care being taken to exclude any underlying tissue.^52^ We will use the sum of four-skinfold thickness to record measurements. All the research staff have been trained by experts and will be evaluated with the support of collaborators from St Johns Hospital and Research Institute, Bangalore. We will also explore to study weight for length and waist girth in centimetres as coprimary outcomes.

### Assessment of other covariates

We want to confirm whether these parameters are useful in the Indian population. We will obtain information on potential confounders, namely lifestyle factors, age, body mass index (BMI), family history of diabetes, gestational age, parity, medical history, family history of hypertension and socioeconomic status.

### Conduct of study and administration of questionnaire

We plan to use a well-structured interviewer-administered questionnaire for the survey. At first instance, the questionnaire will contain informed consent to participate in the initial screening programme. If the participant agrees to take part, then the questions are followed up further. Extensive literature review and pilot testing will be carried out to develop and design the final format of the questionnaire. We will collect details about:

- Demographic and social characteristics such as age, religion, caste, marital status, profession, education and socioeconomic status.
- We will also collect information on lifestyle-related factors such as dietary intake.
- Physical activity.
- Tobacco use.
- Medical history.

We will collect details of high-risk pregnant women who are positive for any one or more of these traditional risk factors, namely

- Age ≥25 years;
- BMI ≥25 kg/m^2^;
- Family history of diabetes mellitus;
- Any history of stillbirth;
- Gestational diabetes mellitus;
- Gross-congenital anomaly;
- Macrosomia.

All these variables will be used as effect measure modifiers (figure 1).

### Field staff and data collection

**Figure 2:**
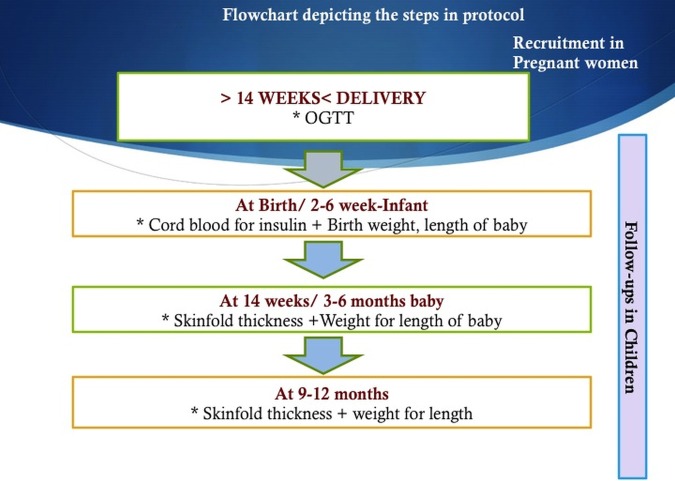
Flow chart depicting the steps in the protocol.

A team of trained research assistants will administer the questionnaire and facilitate implementation of the research protocol. They will be health professionals who are trained in taking blood samples, measuring skinfold thickness and taking height and weight measurements. The team will be stationed in the hospital and would facilitate implementation of the protocol under the supervision of the research associate. During their visit to the hospital, pregnant women will be administered the questionnaire and the team will collect blood specimens as per protocol at the 24–28th week. The team will collect the samples of children and the skinfold measurements will be measured when the children are brought to the paediatric outpatient department (OPD) for immunisation. The schedule of immunisation is at birth, 6, 10 and 14 weeks of age (2.5–3 months). The first follow-up measurement will be made when the children are brought to the OPD at 14 weeks of age for the third dose of polio and diphtheria, pertussis and tetanus. For children who miss this visit, the health workers will visit their house to complete the follow-up examination and collect blood samples within a month of the missing visit. The parents would be informed that the second follow-up is scheduled at 6 months of age, at the hospital, and that they should bring the children to the hospital again at that time. Through this, we will also be testing the feasibility of collecting blood samples from children for glucose, insulin and others (see figure 2).

### Plan for bias minimisation and analysis

Adequate power is essential in a cohort study and we have powered our study based on conservative estimates of sustained and clinically meaningful differences in clinical outcome. The participation of volunteers might indicate that they are more willing to self-select. However, this will be minimised, as we will select geographically and socioeconomically diverse health centres in the city of Bangalore.

In order to control for potential confounding of the association between covariates and hyperglycaemia, we will examine a large number of variables simultaneously. For this purpose, multivariate regression analysis will be used. Variable selection will be based primarily on prior knowledge and also the outcome of crude analysis. Variables with a p value >0.20 in the univariate analysis will be included in the multivariate analysis. Possible interactions will also be explored by including product terms in the model. Multivariate logistic regression analysis will be applied for calculation of risk for various maternal risk factors.

At the initial screening during analysis, a summary measure will be obtained by computing the weighted average of the ratios, where the weights are the inverses of the variances of the stratum-specific prevalence ratios. An approximation of the variance of a prevalence difference as described in the standard literature will be used to calculate the summary prevalence difference based on the formulae described. We will look at the variables associated with the presence of risk factors. Crude ratios (unadjusted), p value and 95% CI will be calculated to evaluate possible associations between covariates and outcome variables.

Loss to follow-up may bias the study if it is related to the outcome of our study. We plan to control this through our analysis, by collecting some information from the centres and people are who might potentially be lost to follow-up. Also, measurement error is unlikely due to the rigorous standardised measures of exposure (hyperglycaemia) and outcome (adiposity measured by Holtain's calipers). There is no scope for differential misclassification of the outcome, as the research assistants who measure adiposity do not have access to the results of the exposure. Hence, any misclassification of the outcome will only be non-differential. Centralised training, quality checks and use of standard equipments will minimise the misclassification, despite being non-differential.

### Plan for statistical analysis

We will calculate indicators addressing logistical feasibility and the process of recruitment in public health facilities in India. We will also calculate the risk of diabetes in children at birth, 3 and 6 months of follow-up as a result of hyperglycaemia in the mother. We will compare the adiposity levels up in infants and their association with maternal glycaemic levels. At the initial screening, a summary measure will be obtained by computing the weighted average of the ratios, where the weights are the inverses of the variances of the stratum-specific prevalence ratios. An approximation of the variance of a prevalence difference as described in the standard literature will be used to calculate the summary prevalence difference based on the formulae described.^53^ We will look at the variables associated with the presence of risk factors. Crude prevalence, OR, p value and 95% CI will be calculated to evaluate possible associations between covariates and outcome variables.

In order to control for potential confounding of the association between covariates and hyperglycaemia, we will examine a large number of variables simultaneously. For this purpose, multivariate regression analysis will be used. Variable selection will be based primarily on prior knowledge and also the outcome of crude analysis. Variables with a p value >0.20 in the univariate analysis will be included in the multivariate analysis. Possible interactions will also be explored by including product terms in the model.^53^ All parameters will be analysed for statistical significance by using Pearson’s χ^2^ test and the analysis of variance test. Multivariate logistic regression analysis will be applied for calculation of risk for various maternal risk factors. A p value less than 0.05 will be considered as statistically significant. For analytical purposes, patients will be grouped according to glucose levels and OGTT values and high-risk characteristics.

### Ethical considerations

All participants are required to provide written informed consent (see online supplementary appendix).

## Discussion

In summary, previous studies in selected populations suggest that hyperglycaemia in pregnancy may be an important risk factor for obesity and type 2 diabetes in their offspring. Whether the same effects are seen in Indian populations and at degrees of glycaemia which are less severe than hyperglycaemia is not known. On the basis of the model proposed in the protocol here, this knowledge may be extremely important for developing an effective strategy for the control of the obesity-hyperglycaemia epidemic in low-income and middle-income countries. The results from our study can provide insights into the relation of adiposity during infancy and childhood into adult adiposity and T2DM.^54^

Considerable evidence has emerged from the ‘Mysore Parthenon Study’,^55^ ^56^ including the background on the hypothesis, U-shaped birthweight diabetes association^57–60^ and higher insulin resistance in adipose children.^59^ There is considerable evidence on the effect of gestational diabetes mellitus and hyperglycaemia during pregnancy.^30^ ^61–65^ The current public health scenario in India is marked by limited accomplishments and persisting major public health problems.^66^ Pilot studies such as ours will pave the way for planning large cohort studies and provide credible information for evidence-based public health planning and decision-making in India.^67^ We aim to study the feasibility of conducting a large cohort study through the current study. The results from our study will provide inputs specifically to test the logistics of the study methodology, feasibility of doing some tests such as cord-blood serum c-peptide, glucose testing in infants and understanding recruitment issues in addition to mobility of pregnant women and determinants of following them up successfully and viability of following pregnant women in a diverse background.

## References

[1] Prasad A Type 2 diabetes mellitus in young: need for early screening. Indian Pediatr 2011;48:683–8 21992902 10.1007/s13312-011-0111-0

[2] Shaw J Sicree R Zimmet P Global estimates of the prevalence of diabetes for 2010 and 2030. Diabetes Res Clin Pract 2010;87:4–14 19896746 10.1016/j.diabres.2009.10.007

[3] Seshiah V Balaji V Balaji MS Gestational diabetes mellitus in India. J Assoc 2004;52:707–11 15839447

[4] Hales CN Barker DJP The thrifty phenotype hypothesis. Br Med Bull 2001;60:5–20 11809615 10.1093/bmb/60.1.5

[5] Hattersley AT Tooke JE The fetal insulin hypothesis: an alternative explanation of the association of low birthweight with diabetes and vascular disease. Lancet 1999;353:1789–92 10348008 10.1016/S0140-6736(98)07546-1

[6] Neel JV Diabetes mellitus: a “thrifty” genotype rendered detrimental by “progress”? Am J Hum Genet 1962;14:353–62 13937884 PMC1932342

[7] Fall CHD Non-industrialised countries and affluence. Br Med Bull 2001;60:33–50 11809617 10.1093/bmb/60.1.33

[8] Alcolado J Thomas A Maternally inherited diabetes mellitus: the role of mitochondrial DNA defects. Diabet Med 1995; 12:102–8 7743754 10.1111/j.1464-5491.1995.tb00438.x

[9] Dabelea D The diabetic intrauterine environment: short and long-term consequences. *Gestational Diabetes During and After Pregnancy*. London: Springer, 2010:227–39

[10] Poston L Developmental programming and diabetes—the human experience and insight from animal models. Best Pract Res Clin Endocrinol Metab 2010;24:541–52 20832735 10.1016/j.beem.2010.05.007

[11] Fall CHD Evidence for the intra-uterine programming of adiposity in later life. Ann Hum Biol 2011;38:410–28 21682572 10.3109/03014460.2011.592513 PMC3428869

[12] Newsome C Shiell A Fall C Is birth weight related to later glucose and insulin metabolism?—A systematic review. Diabet Med 2003;20:339–48 12752481 10.1046/j.1464-5491.2003.00871.x

[13] Fall C Stein C Kumaran K Size at birth, maternal weight, and type 2 diabetes in South India. Diabet Med 1998;15:220–7 9545123 10.1002/(SICI)1096-9136(199803)15:3<220::AID-DIA544>3.0.CO;2-O

[14] Dabelea D Knowler WC Pettitt DJ Effect of diabetes in pregnancy on offspring: follow-up research in the Pima Indians. J Matern Fetal Neonat Med 2000;9:83–8 10.1002/(SICI)1520-6661(200001/02)9:1<83::AID-MFM17>3.0.CO;2-O 10757442

[15] Pettitt DJ Aleck KA Baird HR Congenital susceptibility to NIDDM. Role of intrauterine environment. Diabetes 1988;37:622–8 3360218 10.2337/diab.37.5.622

[16] Dabelea D Hanson RL Lindsay RS Intrauterine exposure to diabetes conveys risks for type 2 diabetes and obesity: a study of discordant sibships. Diabetes 2000;49:2208–11 11118027 10.2337/diabetes.49.12.2208

[17] Norris SA Osmond C Gigante D Size at birth, weight gain in infancy and childhood, and adult diabetes risk in five low- or middle-income country birth cohorts. Diabetes Care 2012;35:72–9 22100968 10.2337/dc11-0456 PMC3241316

[18] Inturrisi M Lintner NC Sorem KA Diagnosis and treatment of hyperglycemia in pregnancy. Endocrinol Metab Clin North Am 2011;40:703 22108276 10.1016/j.ecl.2011.09.002

[19] Kjos SL Buchanan TA Gestational diabetes mellitus. N Engl J Med 1999;341:1749–56 10580075 10.1056/NEJM199912023412307

[20] Tripathi R Tolia N Gupta VK Screening for gestational diabetes mellitus: a prospective study in a tertiary care institution of North India. J Obstetr Gynaecol Res 2012;38:351–7 10.1111/j.1447-0756.2011.01706.x 22176476

[21] Jovanovic L Pettitt DJ Gestational diabetes mellitus. JAMA 2001;286:2516–18 11722247 10.1001/jama.286.20.2516

[22] Torloni MR Betrán AP Horta BL Prepregnancy BMI and the risk of gestational diabetes: a systematic review of the literature with meta-analysis. Obes Rev 2009;10:194–203 19055539 10.1111/j.1467-789X.2008.00541.x

[23] Brochier ML Arwidson P Coronary heart disease risk factors in women. Eur Heart J 1998;19:A45–52 9519343

[24] Krishnaveni GV Hill JC Veena SR Gestational diabetes and the incidence of diabetes in the 5 years following the index pregnancy in South Indian women. Diabetes Res Clin Pract 2007;78:398–404 17640759 10.1016/j.diabres.2007.06.002 PMC2358951

[25] Jones CW Gestational diabetes and its impact on the neonate. Neonatal Netw 2001;20:17–23 12144115 10.1891/0730-0832.20.6.17

[26] Kim C Newton KM Knopp RH Gestational diabetes and the incidence of type 2 diabetes. Diabetes Care 2002;25:1862–8 12351492 10.2337/diacare.25.10.1862

[27] Persson B Hanson U Neonatal morbidities in gestational diabetes mellitus. Diabetes Care 1998;21:B79 9704232

[28] Krishnaveni G Hill J Veena S Truncal adiposity is present at birth and in early childhood in South Indian children. Indian Pediatr 2005;42:527 15995269

[29] Whitaker RC Pepe MS Seidel KD Gestational diabetes and the risk of offspring obesity. Pediatrics 1998;101:e9 9445519 10.1542/peds.101.2.e9

[30] Pettitt DJ Knowler WC Long-term effects of the intrauterine environment, birth weight, and breast-feeding in Pima Indians. Diabetes Care 1998;21:B138 9704241

[31] Vohr BR Lipsitt LP Oh W Somatic growth of children of diabetic mothers with reference to birth size. J Pediatr 1980;97:196–9 7400885 10.1016/s0022-3476(80)80473-2

[32] Silverman B Rizzo T Green O Long-term prospective evaluation of offspring of diabetic mothers. Diabetes 1991;40:121 1748240 10.2337/diab.40.2.s121

[33] Silverman BL Metzger BE Cho NH Impaired glucose tolerance in adolescent offspring of diabetic mothers: relationship to fetal hyperinsulinism. Diabetes Care 1995;18:611–17 8585997 10.2337/diacare.18.5.611

[34] Persson B Gentz J Moller E Follow-up of children of insulin dependent (type I) and gestational diabetic mothers. Growth pattern, glucose tolerance, insulin response, and HLA types. Acta Paediatr Scand 1984;73:778–84 6395625 10.1111/j.1651-2227.1984.tb17775.x

[35] Hollander MH Paarlberg KM Huisjes AJM Gestational diabetes: a review of the current literature and guidelines. Obstetr Gynecol Surv 2007;62:125–36 10.1097/01.ogx.0000253303.92229.59 17229329

[36] Catalano PM Kirwan JP Haugel-de Mouzon S Gestational diabetes and insulin resistance: role in short-and long-term implications for mother and fetus. J Nutr 2003;133:1674S–83S 12730484 10.1093/jn/133.5.1674S

[37] Pettitt DJ. Diabetes in subsequent generations. Diabetes and Pregnancy. An International Approach to Diagnosis and Management. Dornhorst A & Hadden DR, eds. Chichester: John Wiley & Sons, 1996:367–76

[38] Torrens C Brawley L Anthony FW Folate supplementation during pregnancy improves offspring cardiovascular dysfunction induced by protein restriction. Hypertension 2006;47:982–7 16585422 10.1161/01.HYP.0000215580.43711.d1

[39] Group THSCR Hyperglycemia and adverse pregnancy outcomes. N Engl J Med 2008;358:1991–2002 18463375 10.1056/NEJMoa0707943

[40] Rejoice PR Ravishankar AK Differentials in maternal health care service utilization: comparative study between Tamilnadu and Karnataka. World Appl Sci J 2011;14:1661–9

[41] StataCorp L. Stata/SE 10.1. College Station, TX: Stata Corp, 2008

[42] Daniel WW Biostatistics: basic concepts and methodology for the health sciences. John Wiley & Sons, 2010

[43] Kelsey JL Methods in observational epidemiology. USA: Oxford University Press, 1996

[44] Reddy KS India wakes up to the threat of cardiovascular diseases. J Am Coll Cardiol 2007;50:1370–2 17903637 10.1016/j.jacc.2007.04.097

[45] Yusuf S Hawken S Ounpuu S Effect of potentially modifiable risk factors associated with myocardial infarction in 52 countries (the INTERHEART study): case-control study. Lancet 2004;364:937–52 15364185 10.1016/S0140-6736(04)17018-9

[46] Deepa M Farooq S Datta M Prevalence of metabolic syndrome using WHO, ATPIII and IDF definitions in Asian Indians: the Chennai Urban Rural Epidemiology Study (CURES-34). Diabetes/Metab Res Rev 2007;23:127–34 10.1002/dmrr.658 16752431

[47] Dean AG, Sullivan KM, Soe MM. OpenEpi: Open Source Epidemiologic Statistics for Public Health, version 2.3. 1 (updated 23 Jun 2011). http://saber.salud.gob.sv/openepi/Menu/OpenEpiMenu.htm (accessed 23 Apr 2012)

[48] International Association of Diabetes and Pregnancy Study Groups Consensus Panel. International association of diabetes and pregnancy study groups recommendations on the diagnosis and classification of hyperglycemia in pregnancy. Diabetes Care 2010;33:676–82 20190296 10.2337/dc09-1848 PMC2827530

[49] Nesbitt GS Smye M Sheridan B Integration of local and central laboratory functions in a worldwide multicentre study: experience from the Hyperglycemia and Adverse Pregnancy Outcome (HAPO) Study. Clin Trials 2006;3:397–407 17060214 10.1177/1740774506070695

[50] Tanner JM Whitehouse RH Revised standards for triceps and subscapular skinfolds in British children. Arch Dis Child 1975;50:142–5 1130819 10.1136/adc.50.2.142 PMC1544381

[51] Limited H. Holtain Tanner/Whitehouse Skinfold Caliper 2014. http://www.anthropometer.com/tw.php

[52] Geissler C Powers HJ Garrow J Human nutrition. Elsevier/Churchill Livingstone, 2005

[53] Greenland S Rothman KJ Fundamentals of epidemiologic data analysis. Chapter 13. In: Lash TL, ed. Modern epidemiology. 3rd edn Philadelphia: Lippincott Williams & Wilkins, 2008:219

[54] Sachdev HS Fall CH Osmond C Anthropometric indicators of body composition in young adults: relation to size at birth and serial measurements of body mass index in childhood in the New Delhi birth cohort. Am J Clin Nutr 2005;82:456–66 16087993 10.1093/ajcn.82.2.456

[55] Krishnaveni GV Hill JC Leary SD Anthropometry, glucose tolerance, and insulin concentrations in Indian children. Diabetes Care 2005;28:2919–25 16306555 10.2337/diacare.28.12.2919

[56] Hill JC Krishnaveni GV Annamma I Glucose tolerance in pregnancy in South India: relationships to neonatal anthropometry. Acta Obstet Gynecol Scand 2005;84:159–65 15683377 10.1111/j.0001-6349.2005.00670.x

[57] Krishnaveni G Veena S Wills A Adiposity, insulin resistance and cardiovascular risk factors in 9–10-year-old Indian children: relationships with birth size and postnatal growth. J Dev Origins Health Dis 2010;1:403–11 10.1017/S2040174410000498 PMC3272429 22318657

[58] Nightingale C Krishnaveni G Rudnicka A Adiposity and cardiometabolic risk markers among Indian children: comparison with Indian and white European children in the UK. J Epidemiol Commun Health 2011;65:A16

[59] Barr J Veena S Kiran K The relationship of birthweight, muscle size at birth and post-natal growth to grip strength in 9-year-old Indian children: findings from the Mysore Parthenon study. J Dev Orig Health Dis 2010;1:329–37 23750316 10.1017/S2040174410000309 PMC3672832

[60] Kehoe S Krishnaveni G Lubree H Prediction of body-fat percentage from skinfold and bio-impedance measurements in Indian school children. Eur J Clin Nutr 2011;65:1263–70 21731039 10.1038/ejcn.2011.119 PMC3242049

[61] Silverman BL Rizzo TA Cho NH Long-term effects of the intrauterine environment. The Northwestern University Diabetes in Pregnancy Center. Diabetes Care 1998;21:B142 9704242

[62] Sobngwi E Boudou P Mauvais-Jarvis F Effect of a diabetic environment in utero on predisposition to type 2 diabetes. Lancet 2003;361:1861–5 12788573 10.1016/S0140-6736(03)13505-2

[63] Gillman MW Rifas-Shiman S Berkey CS Maternal gestational diabetes, birth weight, and adolescent obesity. Pediatrics 2003;111:e221–6 12612275 10.1542/peds.111.3.e221

[64] Whitaker RC Pepe MS Seidel KD Gestational diabetes and the risk of offspring obesity. Pediatrics 1998;101:e9 9445519 10.1542/peds.101.2.e9

[65] Bhargava SK Sachdev HS Fall CHD Relation of serial changes in childhood body-mass index to impaired glucose tolerance in young adulthood. N Engl J Med 2004;350:865–75 14985484 10.1056/NEJMoa035698 PMC3408694

[66] Babu GR Laxminarayan R The unsurprising story of MDR-TB resistance in India. Tuberculosis 2012;92:301–6 . 22504008 10.1016/j.tube.2012.02.009

[67] Babu GR ‘Opportunities for improving public health system in India’ analysis of current state of affairs and pointers for future. *Ann Trop Med Pub Health* 4.2 (2011):69

